# Improved Artificial Protozoa Optimizer-Based PI Control for Temperature and Power Regulation of PCM Thermal Energy Storage

**DOI:** 10.3390/biomimetics11100683

**Published:** 2026-09-22

**Authors:** Cebrail Turkeri

**Affiliations:** 1 School of Engineering, University of Warwick, Coventry CV4 7AL, UK; cebrail.turkeri.2@warwick.ac.uk or cebrail.turkeri@batman.edu.tr; 2 Department of Computer Engineering, Batman University, Batman 72050, Türkiye

**Keywords:** phase change material, thermal energy storage, artificial protozoa optimizer, Lévy flight, discharge regulation

## Abstract

Phase-change material (PCM) thermal energy storage requires effective discharge regulation because the available stored energy and resulting thermal output vary continuously during operation. This study develops an Improved Artificial Protozoa Optimizer (IAPO) for proportional–integral (PI) controller tuning to regulate outlet temperature and released thermal power. IAPO retains the main structure of the original APO while introducing a best-guided Lévy-flight perturbation into the autotrophic and heterotrophic foraging modes. Controller tuning is performed on a nonlinear PCM model previously validated against experimental discharge data, using the integral of time-weighted absolute error as the objective function. For temperature regulation, 25 independent runs show that IAPO reduces the mean ITAE by 6.11% relative to APO and 19.02% relative to Differential Evolution, while exhibiting lower run-to-run variability than APO and DE. Closed-loop comparisons further show the shortest rise and settling times with IAPO for both regulation objectives; for power regulation, the settling time is 11.70% shorter than that obtained with APO. The controller also tracks successive changes in temperature and power references. These results show that the proposed modification improves APO-based PI tuning for the investigated PCM discharge-regulation problems while preserving the simple PI control structure.

## 1. Introduction

The growing emphasis on efficient energy use has increased interest in recovering thermal energy that would otherwise be discharged to the environment. A substantial fraction of primary energy consumption ultimately appears as waste heat, yet its utilization is often limited by mismatches between heat availability and end-use demand in time, temperature level, or location [1]. Thermal energy storage (TES) can partly overcome these mismatches by decoupling heat recovery from subsequent use. Among TES technologies, phase-change material (PCM)-based systems are attractive because latent-heat storage can provide relatively high energy density while maintaining a comparatively narrow temperature range during phase transition [2,3]. PCM storage has therefore been investigated for industrial waste-heat recovery, distributed generation, thermal batteries, and vehicle or fuel-cell thermal management [4,5,6,7]. Tube-based PCM systems have been demonstrated for industrial waste-heat recovery and cascaded utilization of diesel-engine exhaust energy [4,5], while other studies have explored PCM-integrated heat exchangers [6] and fuel-cell waste-heat recovery for preheating [7]. Recent studies have also investigated heat-transfer enhancement strategies for PCM-based thermal storage, including wavy-fin and nano-enhanced configurations for accelerating PCM melting and tapered crossbar-fin arrangements with dual nano-enhanced PCMs for improving solidification performance [8,9]. These applications highlight the need not only to store thermal energy effectively but also to deliver it in a form that meets downstream requirements.

During discharge, the thermal state of a PCM storage system changes continuously, and the relationships among heat-transfer-fluid flow rate, stored energy, outlet temperature, and released thermal power can be nonlinear. Maintaining a prescribed thermal output therefore requires an appropriate control strategy as the available stored energy decreases [10,11]. Model predictive control (MPC) offers prediction and explicit constraint handling and has recently been applied to a PCM thermal storage device at an industrially relevant scale [12]. Artificial intelligence (AI)-assisted MPC and other data-driven methods have also been investigated for TES prediction, operation, and control [13,14,15]. CFD coupled with machine learning models has also been applied to PCM thermal systems, including the prediction of melt-fraction behaviour in finned hybrid PCM cold-energy storage configurations [16]. Such approaches, however, depend on the quality of the plant or data-driven model and may become more demanding as system complexity and constraints increase [10,11,12,13,14,15]. Proportional–integral (PI) and proportional–integral–derivative (PID) control remain attractive when a simpler and more transparent feedback structure is preferred. Zhang et al., for example, regulated the outlet temperature of a latent-heat storage system by adjusting working-fluid inlet velocity through PID control [17]. Studies on related thermal processes likewise show that controller performance is sensitive to process dynamics, uncertainty, and parameter selection [18].

This sensitivity has encouraged optimization-based PI/PID tuning. Rather than relying only on empirical rules, numerical optimization can select controller gains according to a closed-loop performance criterion while respecting prescribed parameter bounds. Metaheuristic methods are suitable for such derivative-free problems because they do not require analytical gradients and can be applied directly to simulation-based objective functions [19]. Their performance nevertheless depends on the search mechanism, objective function, computational budget, parameter bounds, and controlled plant [19]. In parallel, AI and evolutionary optimization have become increasingly relevant to TES for prediction, design, operational optimization, and control [3,14,15,20]. Recent reviews emphasize that these methods should be assessed not only through optimization metrics but also through application-specific engineering performance [3,20]. Genetic optimization has also been used directly in PCM thermal storage design [21]. These developments motivate the investigation of newer bio-inspired optimizers for systematic PI gain tuning in PCM discharge regulation.

One such method is the Artificial Protozoa Optimizer (APO), which models autotrophic and heterotrophic foraging together with dormancy and reproduction behaviours [22]. APO provides a suitable basis for the present tuning problem because it performs population-based search over bounded continuous decision variables and incorporates distinct mechanisms for exploration and exploitation through its foraging, dormancy, and reproduction behaviours [22]. Its original formulation involves only two algorithm-specific parameters and has demonstrated competitive performance in constrained continuous engineering design problems [22]. These characteristics are useful for simulation-based PI tuning, where each candidate gain pair is evaluated directly through the nonlinear closed-loop PCM model without requiring analytical gradient information.

Since its introduction, several variants have been proposed for different optimization tasks. Lai et al. developed an improved APO for photovoltaic-model parameter estimation [23], while other studies introduced a phototaxis-inspired search for CNN architecture optimization [24] and combined diversification and refinement strategies for engineering optimization and feature selection [25,26]. Pan et al. further incorporated Gaussian and Lévy mechanisms into a Taguchi-assisted APO for multilevel image segmentation [27], showing that Lévy flight has already been used within APO variants. More generally, Lévy flights are widely used in stochastic optimization because their heavy-tailed step distributions permit frequent short movements together with occasional long-range transitions [28,29]. Optimization-based tuning of PI-type controllers has also been reported for nonlinear control applications such as centrifugal-fan pressure regulation [30]. However, no study was identified in the literature reviewed here that uses an APO-based PI-tuning framework for PCM discharge control while addressing both outlet-temperature and released-thermal-power regulation. This motivates a targeted APO modification for the two control objectives considered here.

Accordingly, this study develops an Improved Artificial Protozoa Optimizer (IAPO) for PI gain tuning in PCM thermal energy storage. The original APO structure is retained, while a best-guided Lévy-flight perturbation is added to the autotrophic and heterotrophic foraging modes; dormancy and reproduction remain unchanged. The method is applied to a nonlinear PCM model previously validated against experimental discharge data, with separate PI gains determined for outlet-temperature and released-thermal-power regulation by minimizing the integral of time-weighted absolute error (ITAE). The proposed modification is examined directly against the original APO [22], with Differential Evolution (DE) used as an established evolutionary benchmark [31]. Controller-level performance is additionally compared with the classical Tyreus–Luyben PI-tuning method [32] and ARO-based controller-tuning results previously reported for the same PCM system [33]. Repeated-run statistics and convergence behaviour are evaluated for temperature tuning, followed by closed-loop and varying-reference tests for both regulation objectives. IAPO yields lower repeated-run ITAE values than APO, DE, and ARO for temperature tuning and shorter transient times in the examined closed-loop comparisons, with a more pronounced improvement over APO in power regulation.

## 2. PCM Thermal Storage System and Control-Oriented Model

### 2.1. System Configuration and Control Variables

The plant considered in this study is a PCM-based thermal storage unit integrated with a diesel-engine distributed energy system. Related tube-type PCM storage configurations for industrial and diesel-engine waste-heat recovery have previously been reported in [4,5]. For the present controller-design study, the nonlinear discharging model described below is used as the plant representation. Its parameter set was obtained from previously acquired experimental discharging data; no additional experimental campaign or parameter-identification procedure is performed in this work.

The installation comprises a 200 kW-level diesel engine and a tube-based PCM storage module designed to recover heat from the engine exhaust. The storage module contains 124 vertically oriented steel tubes arranged in a staggered configuration. The PCM considered in this study is the carbonate-based PCM used in the high-temperature storage module of the experimental system [5,33]. It is enclosed between the inner and outer tube walls, while the internal passage serves as the discharging channel. During charging, high-temperature exhaust gas passes across the external tube surfaces and transfers heat to the steel walls and PCM. During discharging, lower-temperature air flows through the internal channels and extracts the stored thermal energy. The physically separated flow paths prevent direct contact between the discharging air and the exhaust gas. The configuration of the storage module and the corresponding charging and discharging paths are illustrated in Figure 1. The nine locations marked I–IX in Figure 1 represent the thermocouple positions used to determine the average module temperature; I–III, IV–VI, and VII–IX correspond to the front, middle, and back measurement locations along the flow direction, respectively [5,33].

For control purposes, the inlet volumetric air-flow rate, ${\dot{V}}_{air}$, is used as the manipulated variable. Depending on the control objective, the regulated output is either the outlet air temperature, $T_{out}$, or the released thermal power, $P_{out}$. The thermal state of the storage unit is represented by the average module temperature,

(1) $$T_{mod}\left(t\right)=\frac{1}{N_{s}}\sum_{k=1}^{N_{s}}T_{k}\left(t\right),\;\;\;\;\;\;\;\;N_{s}=9,\;$$

where $T_{k}$ denotes the temperature recorded at the k-th measurement position. The nine measurement positions correspond to those used in the experimental characterization of the storage module. The main physical and thermophysical parameters used in the simulation model are listed in Table 1 and are adopted from the experimentally investigated PCM storage module reported in [5,33].

The nominal phase-change temperature of the PCM is 760 K. This material property should be distinguished from $T_{cut}$, which denotes the average module temperature used to switch between the latent- and sensible-regime equations of the lumped model.

### 2.2. Nonlinear Discharging Model

The discharging process is represented by a lumped, semi-empirical thermal model. Separate equations are used for the latent and sensible regimes because the equivalent thermal capacity and identified heat-transfer coefficients differ between the two operating regions. The average module-temperature dynamics are expressed as

(2) $$\left\{\begin{array}{c} \left[m_{PCM}c_{PCM,l}+m_{tube}c_{tube}+m_{PCM}c_{h}\right]\frac{dT_{mod}}{dt}=-h_{lat}A\left(T_{mod}-T_{in}\right),\;\;T_{mod}>T_{cut},\\ \left[m_{PCM}c_{PCM,s}+m_{tube}c_{tube}\right]\frac{dT_{mod}}{dt}=-h_{sens}A\left(T_{mod}-T_{in}\right),\;\;\;\;\;\;\;\;\;\;\;T_{mod}\leq T_{cut},\; \end{array}\right.\;$$

where $T_{in}$ is the inlet-air temperature, $h_{lat}$ and $h_{sens}$ are the mean convective heat-transfer coefficients in the latent and sensible regimes, respectively, and $c_{h}$ is the equivalent specific heat contribution associated with the latent heat:

(3) $$c_{h}=\frac{H_{PCM}}{T_{sp}-T_{ep}}\cdot$$

The total cross-sectional area of the 124 release channels is

(4) $$A_{c}=N_{tube}\frac{\pi d_{i}^{2}}{4},$$

and the mean inlet-air velocity is calculated from the volumetric flow command according to

(5) $$\upsilon =\frac{{\dot{V}}_{air}/3600}{A_{c}},$$

where ${\dot{V}}_{air}$ is expressed in m^3^ h^−1^ and υ in m s^−1^. The convective heat-transfer coefficient is represented by

(6) $$h_{j}\left(\upsilon \right)=\upsilon^{n}{\left(SF\right)}_{j},\;j\in \left\{lat,sens\right\},$$

where n is the fixed exponent used in the previously identified model, and ${\left(SF\right)}_{j}$ is the regime-dependent coefficient combining the effects of the channel geometry and relevant air properties. A heat-release coefficient is introduced to represent the fraction of heat transferred from the PCM and steel tube to the discharging air:

(7) $$\eta_{j}=\frac{Q_{air}}{Q_{PCM}+Q_{tube}}=r_{1,j}\frac{\upsilon }{T_{mod}}+r_{2,j},$$

where $Q_{PCM}$ and $Q_{tube}$ denote the heat released by the PCM and steel tube, respectively, and $Q_{air}$ is the heat absorbed by the air.

The coefficient $r_{2,lat}$ is constant in the latent regime. In the sensible regime, $r_{2,sens}$ is calculated from the balance condition imposed at the transition temperature $T_{cut}$ = 650 K. This treatment preserves the transition between the two identified model regions without introducing an independently fitted sensible-regime value of $r_{2}$.

The outlet air temperature is obtained using the air-side relation adopted in the original model,

(8) $$Q_{air}=m_{air}c_{p,0}\left(T_{out}-T_{in}\right),$$

where $m_{air}$ is the effective air mass associated with a representative release channel. In the Simulink implementation,

(9) $$m_{air}=\rho_{air}\left(\frac{A_{c}}{N_{tube}}\right)L_{tube}\cdot$$

The identified coefficients retained in the simulation model are summarized in Table 2.

The coefficients in Table 2 are retained from the previously identified model and are not re-estimated in this study.

For the power-regulation problem, the mass-flow rate of the complete air stream is calculated as

(10) $${\dot{m}}_{air}=\rho_{air}\left(\frac{{\dot{V}}_{air}}{3600}\right)\cdot$$

The released thermal power is subsequently determined from the increase in air enthalpy:

(11) $$P_{out}={\dot{m}}_{air}\left[c_{p}\left(T_{out}\right)T_{out}-c_{p}\left(T_{in}\right)T_{in}\right]$$

The temperature-dependent air specific heat used in the Simulink power calculation is

(12) $$c_{p}\left(T\right)=0.93972+1.96727\times 10^{-4}T$$

where T is expressed in K and $c_{p}$ in kJ kg^−1^ K^−1^. Equation (11) therefore gives $P_{out}$ in kW.

### 2.3. Prior Model Validation and Operating Conditions

The parameters of the nonlinear discharging model were previously identified from experimental data using a multi-population genetic algorithm. The experimental dataset comprised five discharging cases evaluated over a 5000 s test period, with initial module temperatures ranging from 405 to 681 K, inlet-air-flow rates from 400 to 575 m^3^ h^−1^, and average inlet-air temperatures from 360 to 518 K, thereby covering both latent and sensible operating conditions [33]. Nine thermocouples installed at different tube locations were used to determine the average module temperature, while the outlet air temperature was measured directly [33]. The regime-dependent model coefficients listed in Table 2 were identified from these data and are retained without re-estimation in the present study. Model predictions were subsequently compared with the measured average module and outlet air temperatures over the investigated discharge conditions, giving coefficients of determination greater than 0.999 for $T_{mod}$ and 0.996 for $T_{out}$ [33]. The corresponding predicted–experimental validation profiles for these variables are reported in detail in [33] and are not reproduced here, since the present study uses the previously identified model as the plant for controller tuning rather than repeating the model-development and validation analysis. Released thermal power is subsequently calculated from the air-flow rate and inlet and outlet air temperatures using Equation (11), rather than being independently identified as a separate model state. Measurement uncertainty associated with the experimental platform has also been reported previously [5], with standard uncertainties of 0.5 °C for PCM temperature, 1 °C for heat-transfer-fluid temperature, 0.5% for flow rate, 0.8% for PCM mass, and 0.9% for phase-change enthalpy. Although this experimental identification and validation reduce uncertainty in the nominal plant representation, possible parameter mismatch outside the experimentally investigated operating conditions is not explicitly considered in the present controller-design model.

Unless otherwise stated, the closed-loop simulations are initialized using

(13) $$T_{mod}\left(0\right)=680\;K,\;\;\;\;\;\;T_{in}=300\;K,$$

The initial module temperature of 680 K was selected to represent an experimentally observed mixed latent/sensible thermal state during discharge. In the experimental tests conducted at approximately this average module temperature, the PCM in several monitored tubes had entered the phase-change region, while the remaining monitored locations were still in the sensible regime [33]. The inlet-air temperature was set to 300 K to represent near-ambient air supplied during the discharge process; this value is close to the ambient temperature used in the experimental system and provides a practical baseline for assessing heat release to a relatively cool air stream [5,33]. The volumetric air-flow command is constrained to

(14) $$350\leq {\dot{V}}_{air}\left(t\right)\leq 1350\;m^{3}h^{-1}$$

The proposed optimizer is described in Section 3, while the temperature and power reference values, PI-parameter bounds, objective function, and optimization settings are specified in Section 4.

## 3. Proposed Improved Artificial Protozoa Optimizer

### 3.1. Original Artificial Protozoa Optimizer

The Artificial Protozoa Optimizer (APO), proposed by Wang et al. [22], is a population-based metaheuristic inspired by the foraging, dormancy, and reproductive behaviours of *Euglena*. In APO, each artificial protozoan represents a candidate solution, and the population is iteratively improved through four behavioural operators: autotrophic foraging, heterotrophic foraging, dormancy, and asexual reproduction. The original formulation associates autotrophic foraging and dormancy mainly with exploration, whereas heterotrophic foraging and reproduction mainly contribute to exploitation.

For a minimisation problem with dim decision variables, the position of the i-th protozoan is represented by

(15) $$X_{i}=\left[x_{i}^{1},x_{i}^{2},\cdots ,x_{i}^{dim}\right],\;i=1,2,\cdots ,p_{s},$$

where $p_{s}$ is the population size, dim is the number of decision variables, d is the dimension index, and $x_{i}^{d}$ is the value of the d-th decision variable of $X_{i}$. At the beginning of each iteration, the population is sorted in ascending order of the objective-function values. Accordingly, lower rank indices correspond to better candidate solutions. To avoid the dual use of f in the original formulation, the objective function is denoted here by $F\left(\cdot \right)$, while f is retained for the foraging factor.

#### 3.1.1. Foraging Behaviour

In the autotrophic mode, the i-th protozoan is updated using a randomly selected population member and information obtained from paired neighbours in the ranked population:

(16) $$X_{i}^{new}=X_{i}+f\left[X_{j}-X_{i}+\frac{1}{n_{p}}\sum_{k=1}^{n_{p}}\omega_{a,k}\left(X_{k-}-X_{k+}\right)\right]⨀M_{f},$$

where $X_{i}^{new}$ is the generated trial position, $X_{i}$ is the current position, and $X_{j}$ is a protozoan selected randomly from the population. The parameter $n_{p}$ is the number of ranked-neighbour pairs, while k identifies the current pair. The vectors $X_{k-}$ and $X_{k+}$ are selected from the better- and worse-ranked sides of $X_{i}$, respectively. The scalar $\omega_{a,k}$ weights the contribution of the k-th pair, $M_{f}$ is a binary mapping vector that determines the updated dimensions, and ⨀ denotes element-wise multiplication. At the ranking boundaries, the first- or last-ranked protozoan is reused when the corresponding neighbour is unavailable. The number of neighbour pairs satisfies $1\leq n_{p}\leq \left\lfloor (p_{s}-1)/2\right\rfloor$.

The iteration-dependent foraging factor is calculated as

(17) $$f=rand\left[1+\cos\left(\frac{iter}{{iter}_{max}}\pi \right)\right],$$

where rand is a scalar random number drawn uniformly from [0,1], iter is the current iteration, and ${iter}_{max}$ is the maximum number of iterations. The cosine term progressively reduces the upper bound of the foraging factor as the search proceeds.

The autotrophic neighbour weight is defined by

(18) $$\omega_{a,k}=exp\left[-\left|\frac{F\left(X_{k-}\right)}{F\left(X_{k+}\right)+\varepsilon }\right|\right],$$

where $F\left(X_{k-}\right)$ and $F\left(X_{k+}\right)$ are the objective-function values of the two selected protozoa, and ε is a small positive constant used to prevent division by zero.

In the heterotrophic mode, the trial position is generated around a nearby location while incorporating differences between adjacent members of the ranked population:

(19) $$X_{i}^{new}=X_{i}+f\left[X_{near}-X_{i}+\frac{1}{n_{p}}\sum_{k=1}^{n_{p}}\omega_{h,k}\left(X_{i-k}-X_{i+k}\right)\right]⨀M_{f},$$

where $X_{near}$ is a point generated near $X_{i}$, while $X_{i-k}$ and $X_{i+k}$ are the protozoa whose rank indices are i−k and i+k, respectively. The coefficient $\omega_{h,k}$ is the heterotrophic weight of the k-th neighbour pair. The same boundary treatment used in the autotrophic operator is applied when a neighbour index falls outside the ranked population.

The nearby position is obtained from

(20) $$X_{near}=\left[1\pm \mathrm{Rand}\left(1-\frac{iter}{{iter}_{max}}\right)\right]⨀X_{i},$$

where 1 is a dim-dimensional vector of ones and Rand is a random vector whose elements are independently generated in [0,1]. The positive or negative sign permits the nearby location to be generated on either side of the current solution. In accordance with the original APO implementation [22], the sign is randomly selected as +1 or −1 with equal probability for each heterotrophic update. The factor $1-iter/{iter}_{max}$ contracts the neighbourhood over the iterations.

The heterotrophic weight is calculated as

(21) $$\omega_{h,k}=exp\left[-\left|\frac{F\left(X_{i-k}\right)}{F\left(X_{i+k}\right)+\varepsilon }\right|\right].$$

In both foraging modes, the binary vector $M_{f}$ restricts the update to selected dimensions. For the i-th ranked protozoan, $\left\lceil \dim\;i/p_{s}\right\rceil$ dimensions are activated randomly. Consequently, better-ranked candidates are generally modified in fewer dimensions than lower-ranked candidates.

#### 3.1.2. Dormancy and Reproduction

Dormancy is modelled by replacing the current protozoan with a new candidate generated uniformly within the search bounds:

(22) $$X_{i}^{new}=X_{min}+Rand⨀\left(X_{max}-X_{min}\right),$$

where $X_{min}$ and $X_{max}$ are the lower- and upper-bound vectors, respectively.

The reproduction operator represents binary fission by applying a masked random perturbation to the current position:

(23) $$X_{i}^{new}=X_{i}\pm rand\left[X_{min}+Rand⨀\left(X_{max}-X_{min}\right)\right]⨀M_{r},$$

where $M_{r}$ is the binary reproduction mask. The number of active dimensions in $M_{r}$ is randomly selected as $\left\lceil dim\cdot rand\right\rceil$, and the sign of the perturbation allows the trial position to be generated in either direction from $X_{i}$.

#### 3.1.3. Behaviour Selection and Replacement

At each iteration, only a randomly determined fraction of the population is directed to the dormancy/reproduction branch. This proportion is calculated as:

(24) $$p_{f}=p_{f}^{max}rand,$$

where $p_{f}^{max}$ is the maximum dormancy/reproduction proportion. A total of $\left\lceil p_{s}p_{f}\right\rceil$ population indices are selected randomly for this branch, while the remaining protozoa perform one of the two foraging behaviours.

The probability controlling the selection between autotrophic and heterotrophic foraging is

(25) $$p_{ah}=\frac{1}{2}\left[1+\cos\left(\frac{iter}{{iter}_{max}}\pi \right)\right],$$

where autotrophic foraging is selected when $p_{ah}>rand$; otherwise, heterotrophic foraging is applied. Since $p_{ah}$ decreases with the iteration number, autotrophic foraging is more likely during the earlier search stages.

For protozoa assigned to dormancy or reproduction, the rank-dependent probability is

(26) $$p_{dr}=\frac{1}{2}\left[1+\cos\left(\left(1-\frac{i}{p_{s}}\right)\pi \right)\right],$$

where dormancy is selected when $p_{dr}>rand$; otherwise, reproduction is performed. The value of $p_{dr}$ increases with the rank index, making dormancy more probable for lower-quality protozoa and reproduction more probable for better-ranked candidates.

After a trial solution is evaluated, it replaces the corresponding current protozoan only when it produces a lower objective-function value. The best solution encountered during the search is retained, and the iterative process continues until the prescribed stopping criterion is reached.

The original APO therefore contains two algorithm-specific parameters: the number of neighbour pairs $n_{p}$ and the maximum dormancy/reproduction proportion $p_{f}^{max}$. This baseline formulation is retained in the present study, while the modifications introduced in the proposed version are described in the following subsection.

### 3.2. Proposed Lévy-Flight-Assisted Artificial Protozoa Optimizer

The original APO does not explicitly employ global-best information in its autotrophic and heterotrophic foraging equations. In the proposed Improved Artificial Protozoa Optimizer (IAPO), the original behavioural structure is retained, while a best-guided Lévy-flight perturbation is incorporated into both foraging modes. The added component supplements the candidate generated by the standard APO operator without modifying the dormancy and reproduction mechanisms.

For each protozoan assigned to the foraging branch, a Lévy step vector is generated using Mantegna’s formulation [29]:

(27) $$L_{i}=u_{i}⊘{\left|v_{i}\right|}^{1/\beta },$$

where ⊘ denotes element-wise division and β is the stability index of the Lévy distribution. The random vectors $u_{i}$ and $v_{i}$ are independently generated as

(28) $$u_{i}\sim N\left(0,\sigma_{u}^{2}I\right),\;\;\;\;\;\;\;\;v_{i}\sim N\left(0,I\right),$$

where N denotes the normal distribution and I is the dim×dim identity matrix. In the present implementation, β=1.5, and the scale parameter is calculated as

(29) $$\sigma_{u}={\left[\frac{\Gamma \left(1+\beta \right)\sin\left(\pi \beta /2\right)}{\Gamma \left(\left(1+\beta \right)/2\right)\beta 2^{\left(\beta -1\right)/2}}\right]}^{1/\beta },$$

where $\Gamma \left(\cdot \right)$ denotes the gamma function. The additional perturbation of the i-th protozoan is defined as

(30) $$∆X_{i}^{L}=\lambda L_{i}⊙\left(X_{best}-X_{i}\right),$$

where $X_{best}$ is the best solution available when the update is generated, and λ is the Lévy scaling coefficient. In this study, the Lévy-flight parameters are fixed at β=1.5 and λ=0.01 throughout the study and are not separately tuned for either control problem. The value β=1.5 follows a commonly used setting in Mantegna-type Lévy-flight implementations, while λ=0.01 provides a small scaling of the resulting Lévy perturbation [34]. Keeping these parameters fixed avoids problem-specific tuning of the Lévy component and enables the proposed IAPO modification to be evaluated under the same configuration for both temperature and power regulation. The final foraging candidate is therefore obtained from

(31) $$X_{i}^{new}=X_{i,APO}^{new}+∆X_{i}^{L}$$

where $X_{i,APO}^{new}$ denotes the trial solution generated by the original autotrophic operator in Equation (16) or the original heterotrophic operator in Equation (19). A new Lévy vector is generated independently for each protozoan processed through the foraging branch.

The Lévy component is applied independently of the original foraging mask $M_{f}$. Hence, the rank-dependent mask continues to regulate the standard APO displacement, whereas the additional perturbation may modify the complete decision vector. Lévy flight is not applied to protozoa assigned to dormancy or reproduction. The population initialization, ranking, behavioural-selection rules, dormancy, reproduction, boundary handling, greedy replacement, and global-best update are otherwise retained from the baseline APO. The overall computational sequence of IAPO is illustrated in Figure 2.

## 4. PI Control Design and Optimization Formulation

A conventional PI controller is employed to regulate the discharging behaviour of the PCM thermal storage system. The manipulated variable is the inlet volumetric air-flow rate, ${\dot{V}}_{air}$, whereas the controlled output is either the outlet air temperature, $T_{out}$, or the released thermal power, $P_{out}$. These two regulation objectives are considered separately, and an independent set of PI gains is therefore determined for each control problem.

For a reference signal r(t) and controlled output y(t), the tracking error is defined as

(32) $$e\left(t\right)=r\left(t\right)-y\left(t\right).$$

The PI control law is expressed as

(33) $$u\left(t\right)=K_{p}e\left(t\right)+K_{i}\int_{0}^{t}e\left(\tau \right)d\tau ,$$

where $K_{p}$ and $K_{i}$ are the proportional and integral gains, respectively, and $u\left(t\right)$ represents the commanded inlet-air-flow rate. The control signal is limited to the feasible operating range specified in Equation (14). Unless otherwise stated, the nominal closed-loop simulations use the initial conditions given in Equation (13).

The controller-tuning problem is formulated using the decision vector

(34) $$\theta =\left[K_{p},K_{i}\right].$$

The integral of time-weighted absolute error (*ITAE*) is selected as the objective function:

(35) $$J\left(\theta \right)=ITAE=\int_{0}^{t_{sim}}t\left|e\left(t\right)\right|dt,$$

where $t_{sim}=50$ s is the simulation horizon used during the tuning process. ITAE was selected because its time weighting gives greater importance to errors that persist later in the transient, which is consistent with the objective of achieving rapid and sustained regulation during PCM discharge. Rise time, settling time, and overshoot are evaluated separately as closed-loop performance indicators rather than being incorporated into the tuning objective. Alternative single- or multi-objective formulations, including an explicit control-effort penalty, could lead to different PI gains and performance trade-offs; therefore, the conclusions reported here are specific to the adopted ITAE-based tuning formulation. The optimization problem is therefore formulated as $\underset{\theta }{\min}J\left(\theta \right)$. For outlet-temperature regulation, the PI gains are constrained by $-2.5\leq K_{p}\leq -0.5$ and $-0.8\leq K_{i}\leq -0.05$.

The negative gain range reflects the inverse relationship between the inlet-air-flow rate and outlet temperature under the considered discharge conditions. Increasing the air-flow rate enhances heat extraction from the storage module and generally reduces the outlet air temperature. For thermal-power regulation, the input–output relationship is direct; therefore, positive gain bounds are employed: $0.5\leq K_{p}\leq 2.5$ and $0.05\leq K_{i}\leq 0.8$.

The nominal reference values used for controller tuning are 550 K for outlet-temperature regulation and 90 kW for thermal-power regulation. Additional reference changes are subsequently applied to examine the tracking capability of the IAPO-based PI controller over the investigated operating range.

The proposed IAPO is evaluated against the original APO [22], Differential Evolution (DE) [31], the Tyreus–Luyben (TL) tuning method [32], and ARO-based tuning results [33]. For the stochastic comparison of IAPO, APO, DE, and ARO, each algorithm is independently executed 25 times using a population size of 20 and a maximum of 40 iterations. Identical PI-gain bounds and the same ITAE objective function are used for these algorithms within each regulation problem. The best controller parameters obtained from the tuning procedures are subsequently used for the closed-loop performance comparison. The resulting PI-tuning problem is two-dimensional, since only $K_{p}$ and $K_{i}$ are treated as decision variables. The population size of 20 and maximum of 40 iterations were adopted as a common computational budget for IAPO, APO, DE, and ARO, taking into account the low dimensionality and bounded nature of the search problem. The three stochastic optimizers approach stable objective-function levels within the adopted iteration budget, supporting the adequacy of the 40-iteration termination setting for the present tuning problem. All computation-time measurements were obtained on the same computer and under the same MATLAB/Simulink environment to ensure a consistent comparison among the stochastic optimizers.

## 5. Results and Discussion

The performance of the proposed IAPO-based PI controller is investigated for the two control objectives considered in this study: outlet-temperature regulation and released-thermal-power regulation. The original APO [22] is used as the principal baseline to assess the effect of the proposed modification, while Differential Evolution (DE) [31] is included as a well-established evolutionary optimization method. For the subsequent closed-loop comparison, the classical Tyreus–Luyben (TL) tuning approach [32] is also considered. Artificial Rabbits Optimization (ARO) [33] is included as an additional nature-inspired benchmark in both the repeated-run statistical analysis and the controller-level comparisons. TL is used only in the subsequent closed-loop performance comparisons because its deterministic tuning procedure produces the same controller parameters under identical conditions.

### 5.1. Temperature Regulation

The temperature-regulation problem is first evaluated at the nominal outlet-temperature reference of 550 K. Twenty-five independent runs were used to characterize the run-to-run variability of the stochastic optimizers while keeping the computational cost of the nonlinear closed-loop tuning simulations manageable. This repeated-run evaluation is consistent with the methodology adopted in the original APO study [22], where multiple independent runs were likewise used for statistical assessment. Figure 3 presents the run-wise ITAE values for IAPO, APO, and DE, while Table 3 summarizes the repeated-run statistical results for the four stochastic optimization methods, namely IAPO, APO, DE, and ARO. TL is not included in the repeated-run statistical analysis because its deterministic tuning procedure produces the same controller parameters under identical conditions. In particular, the comparison between IAPO and its original APO counterpart serves as a direct baseline for assessing the effect of the modification introduced in Section 3.2. ARO is also included in the repeated-run statistical assessment, whereas TL is not subjected to repeated-run analysis because its deterministic tuning procedure produces the same controller parameters under identical conditions. Accordingly, the repeated-run statistics in Table 3 are used to characterize the stochastic variability of the optimization outcomes.

IAPO produces the lowest best, worst, and mean ITAE values among the four stochastic algorithms. Its mean ITAE is approximately 6.11% lower than that of APO, 19.02% lower than that of DE, and 5.65% lower than that of ARO. Although ARO gives the lowest standard deviation, IAPO shows lower variability than APO and DE, with standard-deviation reductions of approximately 25.98% and 19.51%, respectively. Thus, under the adopted tuning conditions, the proposed modification improves the average objective value relative to all three stochastic comparison algorithms and improves the run-to-run consistency relative to APO and DE. The pairwise *p*-values for IAPO versus APO, DE, and ARO are all 1.2290 × 10^−5^, which is well below the 0.05 significance level and provides additional statistical support for the observed differences in ITAE. To complement the *p*-value-based significance testing, the magnitude of the differences was also quantified using Cohen’s *d*, calculated using the pooled standard deviation of the 25 independent runs. The absolute Cohen’s *d* values for IAPO relative to APO, DE, and ARO are 3.19, 12.12, and 4.19, respectively, indicating large standardized differences in ITAE relative to the run-to-run variability of the stochastic optimisers. The 95% confidence intervals for the mean ITAE are [1803.61, 1829.39] for IAPO, [1917.38, 1952.22] for APO, [2227.19, 2259.21] for DE, and [1917.36, 1933.24] for ARO.

The average computation times per independent run were 190.45, 187.93, 217.41, and 192.08 s for IAPO, APO, DE, and ARO, respectively. Compared with the original APO, IAPO required an additional 2.52 s per run on average, corresponding to an increase of approximately 1.34%. This relatively small difference indicates that the additional Lévy-flight operation introduces limited computational overhead under the adopted offline tuning conditions, where the overall computational cost is dominated by repeated nonlinear closed-loop simulations.

The corresponding convergence histories are presented in Figure 4. All three methods show a rapid reduction in ITAE during the early iterations. IAPO subsequently reaches a lower objective level than APO and DE and exhibits additional reductions as the search progresses. APO stabilizes at an intermediate ITAE level, whereas DE converges to the highest final objective value among the three methods. This behaviour is consistent with the statistical results in Table 3 and indicates that the modified APO search is able to identify a lower-ITAE solution within the specified iteration budget.

The PI parameters obtained using the different tuning approaches are listed in Table 4. ARO is implemented according to its original formulation [33]. All five methods yield negative proportional and integral gains, consistent with the inverse input–output behaviour of the outlet-temperature control loop discussed in Section 4.

The corresponding time-domain performance indices are summarized in Table 5. The rise-time and settling-time values reported in Table 5 are evaluated using the final PI controller selected from each tuning method. Once the corresponding $K_{p}$ and $K_{i}$ values are fixed, the nonlinear plant model and simulation conditions are deterministic; therefore, these indices are reported as direct closed-loop performance measures rather than repeated-run optimization statistics. IAPO provides the shortest rise-time and settling-time values, 3.6647 s and 7.1417 s, respectively. Relative to APO, these values represent reductions of approximately 1.50% and 1.54%. The improvement is more pronounced relative to DE, for which the reductions are approximately 17.17% and 15.50%, and relative to TL, for which they reach approximately 26.88% and 25.04%. Compared with the ARO-based PI controller, IAPO reduces the rise and settling times by approximately 14.99% and 12.49%, respectively. The overshoot values are very small for all methods, remaining below 0.11%; therefore, the main differences between the controllers are associated with transient duration rather than peak deviation.

From a computational perspective, the wall-clock time required by the classical TL tuning procedure was 44.14 s, compared with an average of 190.45 s per IAPO optimization run under the same computational environment. The lower tuning time of TL reflects its deterministic nature and the absence of repeated population-based candidate evaluations. However, both tuning procedures are performed offline, and the resulting PI controllers retain the same online structure. The additional offline tuning cost of IAPO is therefore accompanied by shorter transient times, with the rise and settling times reduced from 5.0117 to 3.6647 s and from 9.5269 to 7.1417 s, respectively, relative to TL under the nominal temperature-regulation condition.

The closed-loop responses in Figure 5 are consistent with the quantitative results in Table 5. All five PI controllers drive the outlet temperature toward the 550 K reference and reach essentially the same steady operating level. The IAPO-based PI controller reaches the reference earlier, whereas TL, DE, and ARO exhibit comparatively longer transient periods. The difference between IAPO and APO is smaller, which is also consistent with their relatively close time-domain indices. In this nominal temperature-control case, the proposed modification mainly shortens the transient response while retaining the very low overshoot already obtained with the APO-based PI controller.

The tracking performance of the IAPO-based PI controller is further examined under a sequence of changing temperature references, as shown in Figure 6. The controller follows both upward and downward changes in the desired outlet temperature. Short transient deviations occur immediately after several setpoint changes, with larger deviations appearing after the larger downward transitions, but the response subsequently converges to the new reference without sustained oscillation. The result demonstrates satisfactory reference-tracking behaviour over the range examined, beyond the single nominal 550 K operating point.

### 5.2. Power Regulation

The second control objective is regulation of the released thermal power. The nominal reference for the comparative test is 90 kW. In contrast to the temperature-control case, the power-regulation loop exhibits a direct relationship between inlet-air-flow rate and released thermal power within the investigated operating range; accordingly, positive PI gains are obtained for all methods.

Figure 7 compares the closed-loop responses obtained using IAPO, APO, DE, TL, and ARO. All five controllers drive the output toward the 90 kW reference without overshoot. The main difference is observed in the duration of the transient response, with the IAPO-based PI controller approaching the target more rapidly.

The PI gains corresponding to these responses are provided in Table 6. For IAPO, the tuning procedure yields $K_{P}=0.5452$ and $K_{I}=0.7791$ for the 90 kW power-regulation case.

Table 7 presents the time-domain performance indices. IAPO achieves the lowest rise time, 10.2504 s, and the lowest settling time, 19.0326 s. Relative to the original APO, these values are reduced by approximately 12.52% and 11.70%, respectively. Relative to DE, the reductions are approximately 21.16% and 20.52%, while reductions of approximately 32.60% and 32.66% are obtained relative to TL. Compared with the ARO-based PI controller, IAPO reduces the rise time by approximately 16.56% and the settling time by approximately 15.84%. No overshoot is observed for any of the five controllers.

These results indicate a clearer separation between IAPO and the original APO for power regulation than for the nominal temperature-regulation case. Since all methods produce zero overshoot, the improvement obtained with IAPO is reflected primarily in the shorter time required to approach and settle at the desired thermal-power level rather than in a trade-off between speed and overshoot.

Finally, the IAPO-based PI controller is evaluated under successive variations in the power reference. As shown in Figure 8, the controller follows both increasing and decreasing power commands over the investigated operating range. Following each reference change, the released thermal power converges smoothly toward the new target without sustained oscillatory behaviour. This result complements the nominal 90 kW comparison and demonstrates that the controller can track varying power demands within the tested range.

The two control studies show a consistent pattern. For temperature regulation, the repeated-run ITAE results favour IAPO, although the improvement over the original APO in the nominal closed-loop response remains relatively modest. A clearer difference is observed in power regulation, where IAPO provides shorter rise and settling times than all of the compared tuning methods. This difference may be associated with the role of the added Lévy-flight perturbation in the search process. The original APO combines exploration and exploitation through its behavioural operators [22], while subsequent work has shown that further modification of its search mechanism can improve its search ability and convergence performance [23]. The heavy-tailed Lévy component used in IAPO introduces additional search diversification [28], while the best-guided term retains information from the best solution found so far. In the temperature-regulation problem, APO already produces a nominal closed-loop response close to that obtained with IAPO, leaving comparatively limited scope for further improvement at the controller-response level. In contrast, the larger separation between the APO- and IAPO-tuned responses in power regulation suggests that the additional search mechanism was more beneficial for identifying improved PI-gain combinations for this problem. This difference may also be related to the more strongly coupled nonlinear input–output relationship in power regulation, since the released thermal power in Equation (11) depends directly on the air-flow rate and simultaneously on the temperature-dependent enthalpy difference. Moreover, IAPO and APO were evaluated using identical PI-gain bounds, population size, iteration limit, ITAE objective function, simulation horizon, and plant model; thus, their direct comparison allows the effect of the added best-guided Lévy-flight perturbation to be assessed independently of differences in these external optimization settings. The results therefore indicate that the proposed APO modification is beneficial for the PI-tuning problems examined here, while its performance should be interpreted within the validated PCM model, controller structure, search bounds, and operating conditions considered in this study.

From an application perspective, the IAPO-based tuning framework could be considered for other PCM discharge systems in which outlet temperature or released thermal power is regulated through heat-transfer-fluid flow. Potential applications include waste-heat recovery and thermal-battery systems. Since the optimization is performed offline, the use of IAPO does not alter the conventional PI structure implemented online. Its broader applicability would nevertheless require retuning and validation for the PCM, storage configuration, and operating range of each target system.

From a practical implementation perspective, the commanded air-flow rate is constrained to the experimentally relevant operating range of 350–1350 m^3^ h^−1^, as specified in Equation (14). This constraint prevents the controller from requesting air-flow levels outside the considered operating range of the system. However, the present control model does not explicitly represent the dynamic response of the physical inlet-air blower. In a physical implementation, the finite response of the blower and possible limitations on the rate of air-flow variation could affect the transient response following rapid reference changes. These actuator dynamics should therefore be considered in future closed-loop implementation and experimental validation of the proposed controller.

### 5.3. Robustness to Sensor Noise

To examine the closed-loop behaviour under measurement uncertainty, a band-limited white-noise signal was introduced into the outlet-temperature feedback path. The noise power and sample time were set to 0.05 and 0.05 s, respectively, while the nominal temperature reference was maintained at 550 K. As shown in Figure 9, the IAPO-based PI controller remains stable in the presence of the measurement disturbance and maintains the outlet temperature close to the reference after the transient period, with only small fluctuations around the setpoint. This result provides further evidence of the controller’s tolerance to sensor noise under the investigated operating condition.

## 6. Conclusions

This study investigated PI-based temperature and released-thermal-power regulation of a PCM thermal energy storage system using IAPO. The controller design was carried out using a nonlinear plant model identified from experimental discharging data and validated against experimental measurements. The main methodological contribution is the selective incorporation of a best-guided Lévy-flight perturbation into the autotrophic and heterotrophic foraging modes of APO, while the original dormancy and reproduction mechanisms are retained. The resulting IAPO framework was used to tune PI controllers for both outlet-temperature and released-thermal-power regulation. Its performance was assessed against the original APO, DE, the classical TL tuning method, and ARO-based PI control.

For outlet-temperature tuning, IAPO reduced the mean ITAE by 6.11% relative to APO and by 19.02% relative to DE over 25 independent runs. Following the inclusion of ARO in the repeated-run comparison, IAPO also achieved a 5.65% lower mean ITAE than ARO and produced the lowest mean ITAE among the stochastic methods considered. The improvement over APO in the nominal temperature response was comparatively modest, whereas a clearer benefit was obtained for power regulation. In that case, the IAPO-based PI controller reduced the settling time by 11.70% relative to APO and also provided the shortest rise and settling times among the compared controllers. The varying-reference tests further showed that the IAPO-based PI controller tracked both increasing and decreasing temperature and power references over the investigated operating ranges.

The results indicate that the proposed modification is useful for the PI-tuning problems examined in this work, particularly for power regulation. The conclusions are specific to the adopted ITAE-based tuning formulation, validated PCM model, controller structure, parameter bounds, and operating conditions. The additional sensor-noise case further showed that the IAPO-based PI controller maintained stable temperature tracking in the presence of the investigated measurement disturbance. Although the plant model is supported by experimental measurements, the IAPO-based controller has not yet been implemented in a closed loop on the physical storage facility. Future experimental controller validation should further consider the dynamic response of the physical inlet-air blower, measurement noise, time delays, PCM thermophysical-property uncertainties, possible model mismatch, and broader operating conditions.

## Figures and Tables

**Figure 1:**
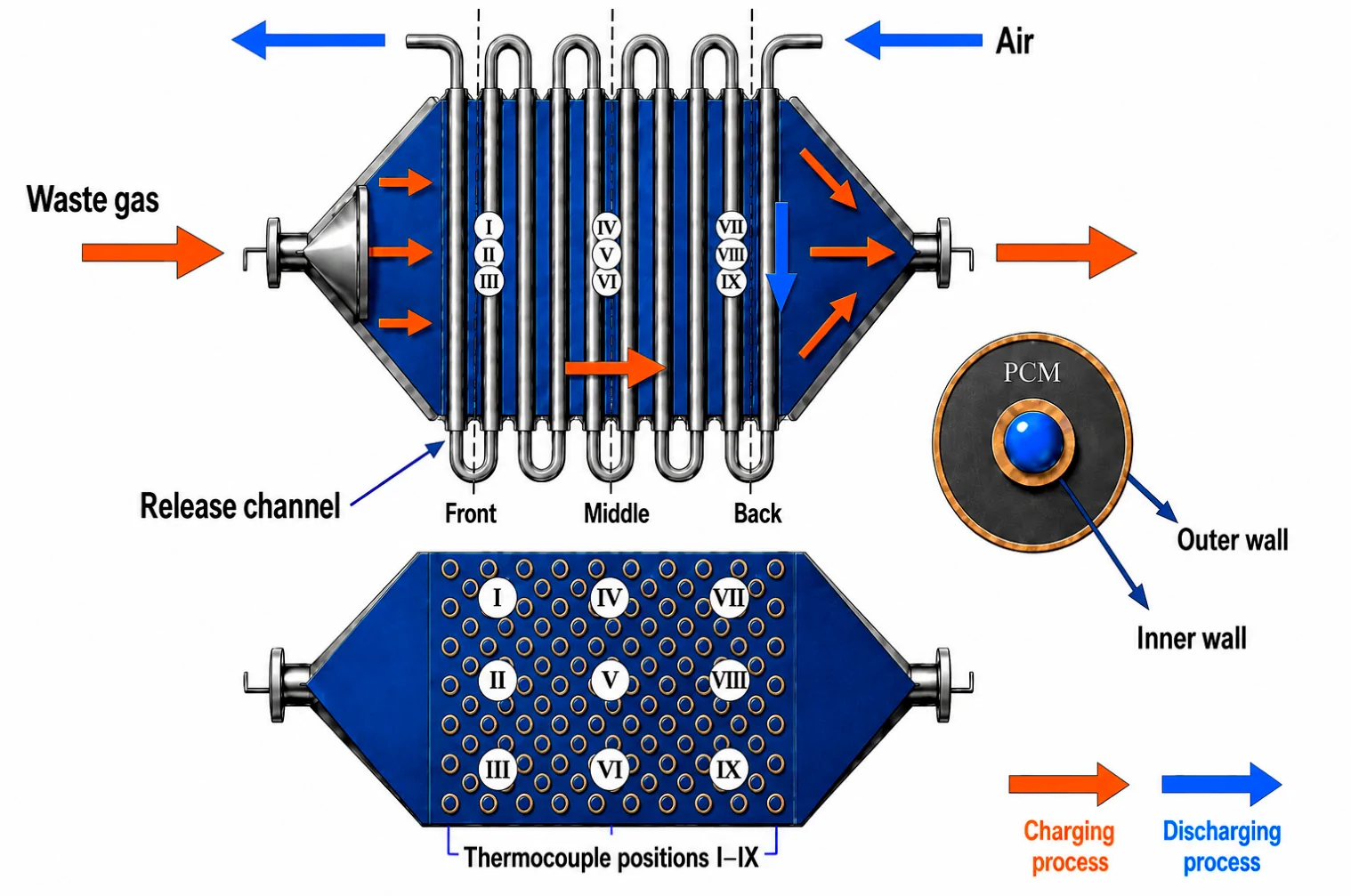
Schematic diagram of the PCM thermal storage module showing the charging and discharging flow paths and thermocouple measurement positions.

**Figure 2:**
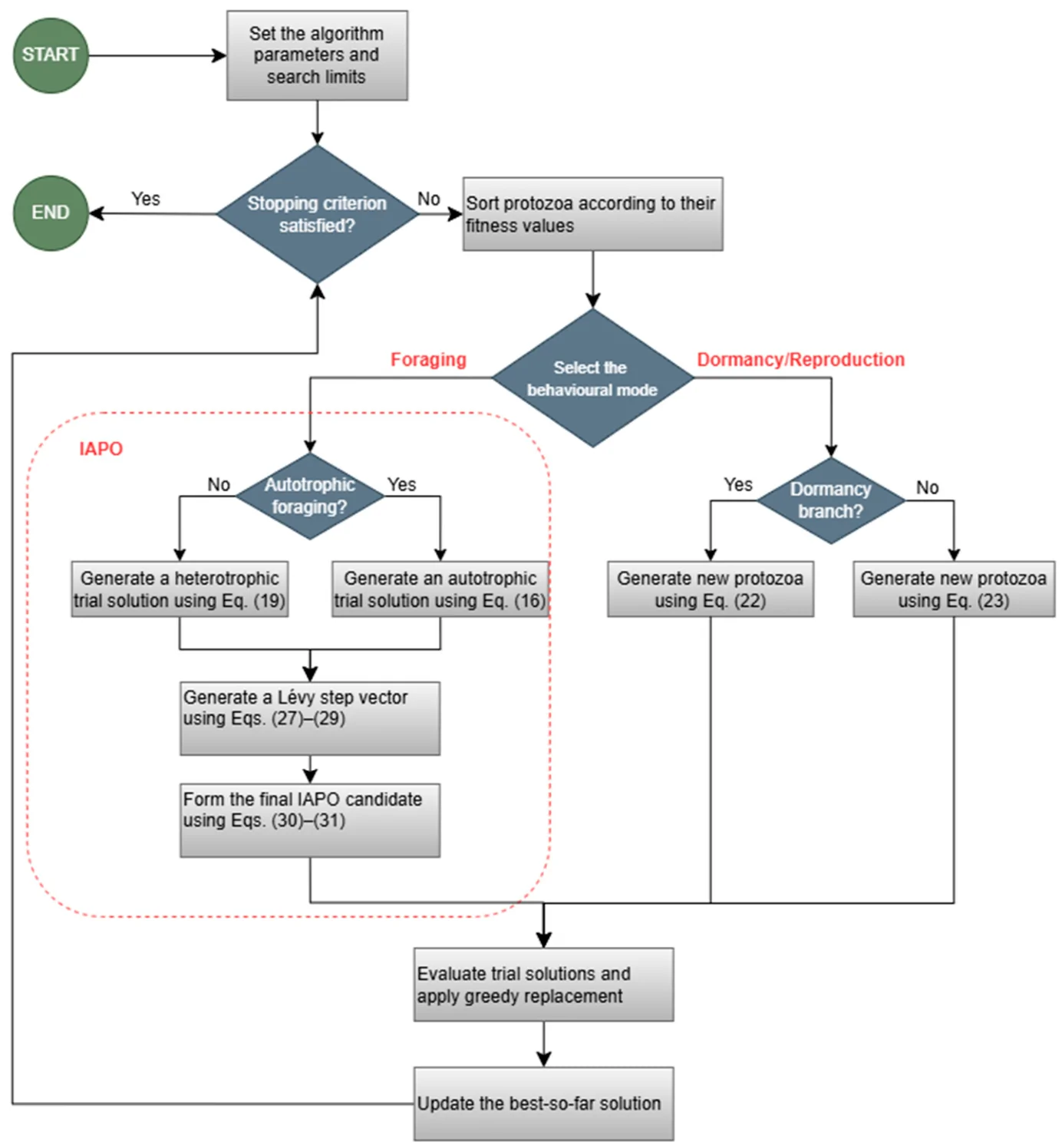
Flowchart of the proposed IAPO.

**Figure 3:**
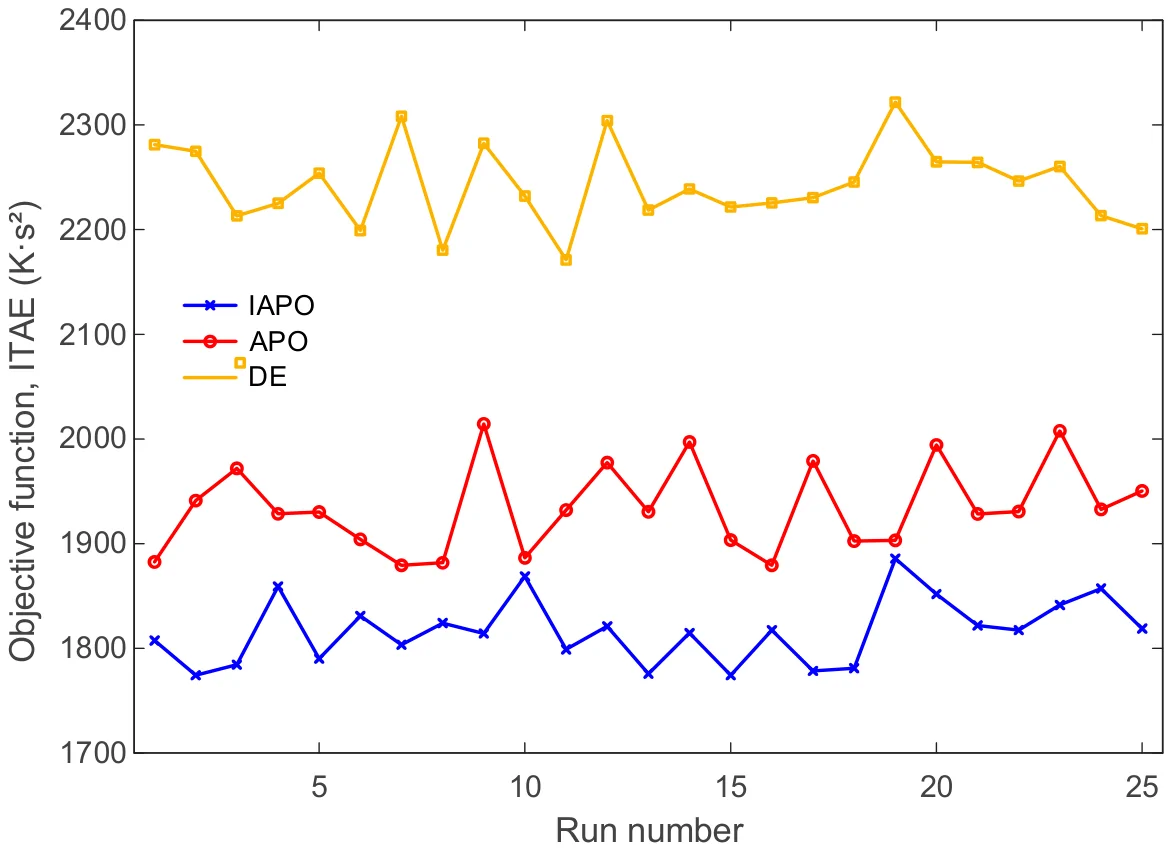
ITAE values obtained over 25 independent runs for outlet-temperature controller tuning.

**Figure 4:**
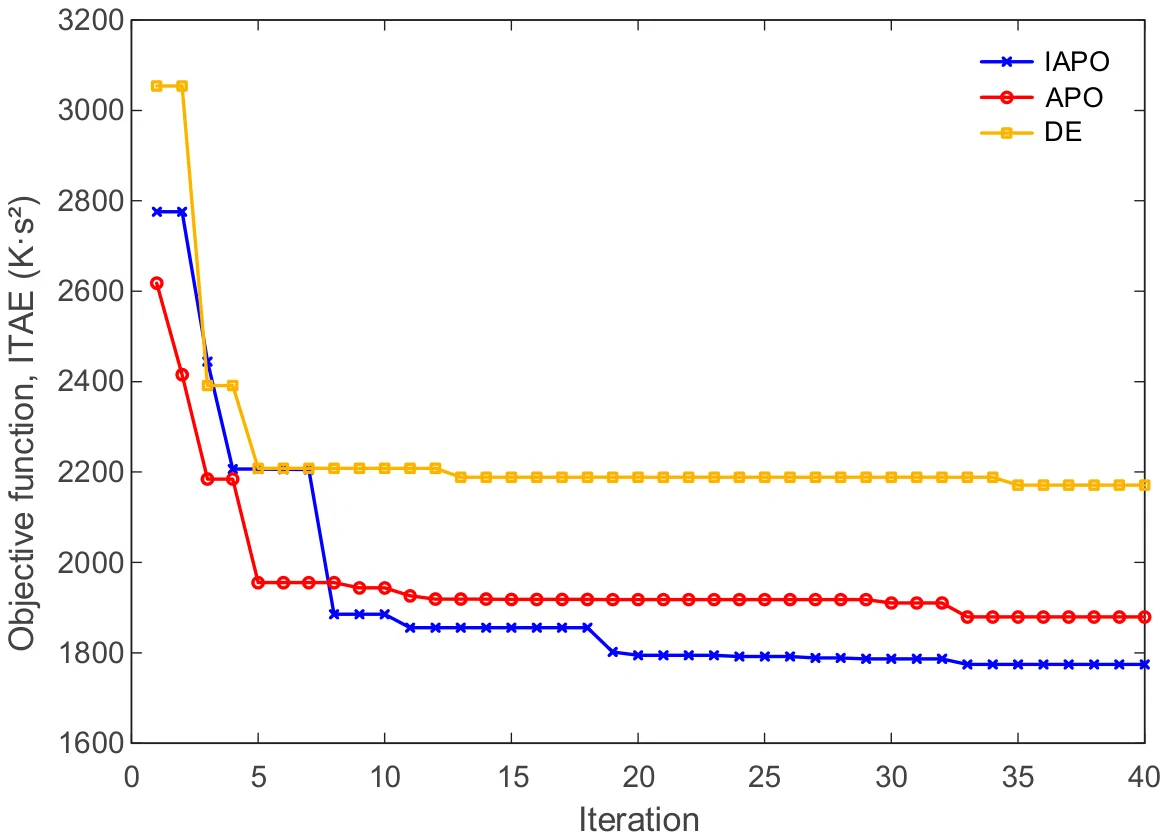
Convergence histories of IAPO, APO, and DE for outlet-temperature controller tuning.

**Figure 5:**
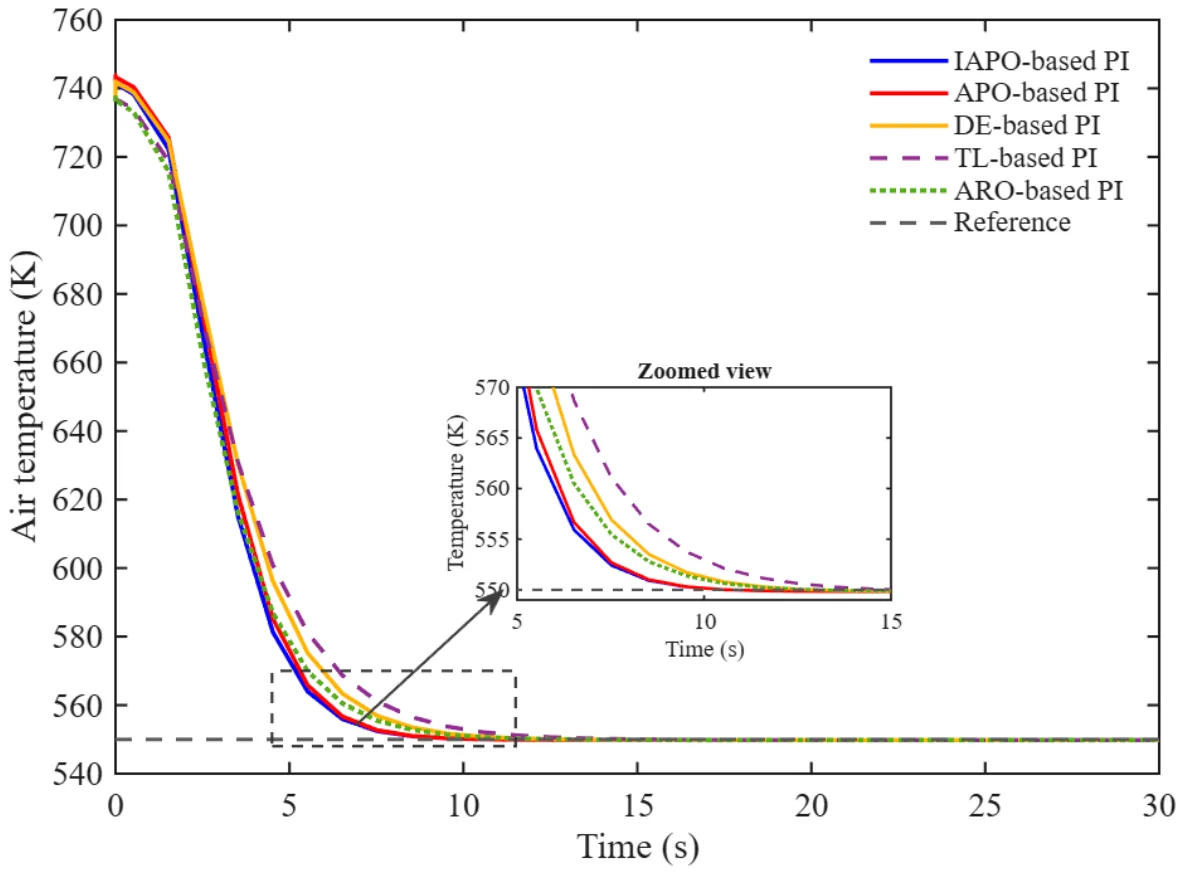
Closed-loop outlet-temperature responses obtained using different PI-tuning methods.

**Figure 6:**
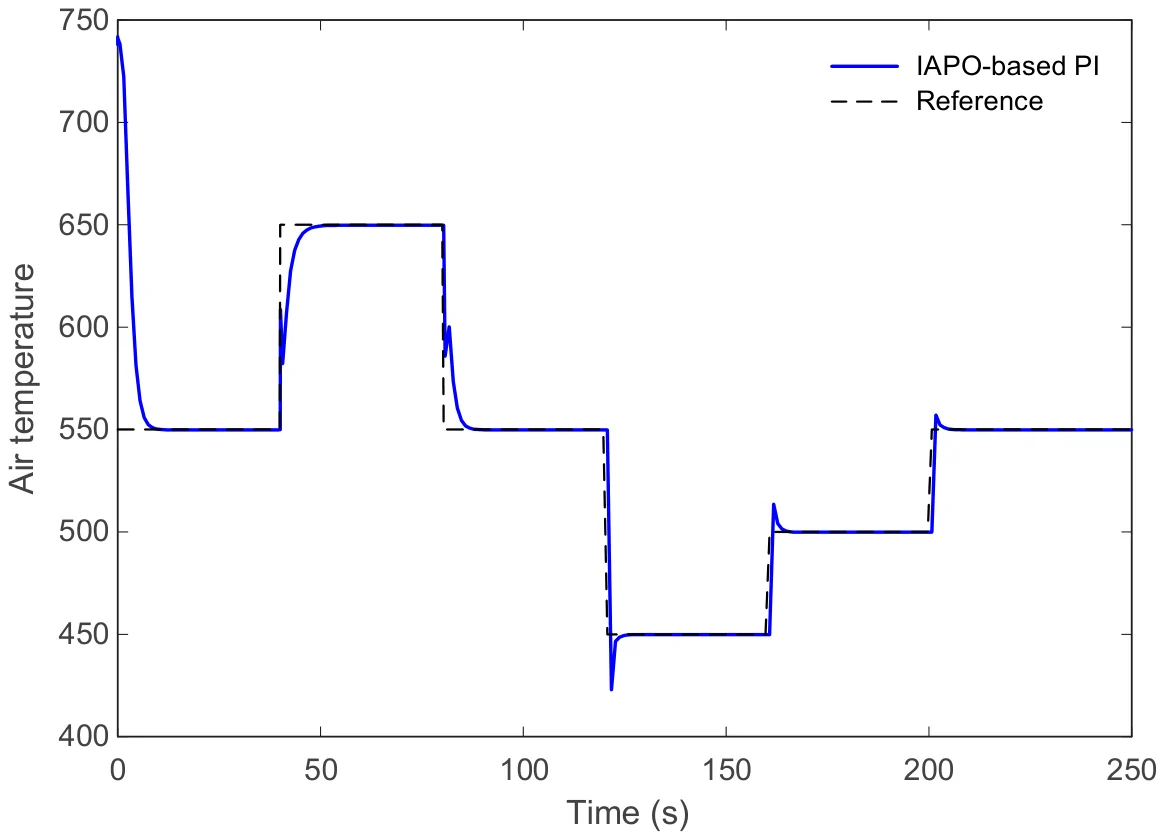
Outlet-temperature tracking of the IAPO-based PI controller under varying reference values.

**Figure 7:**
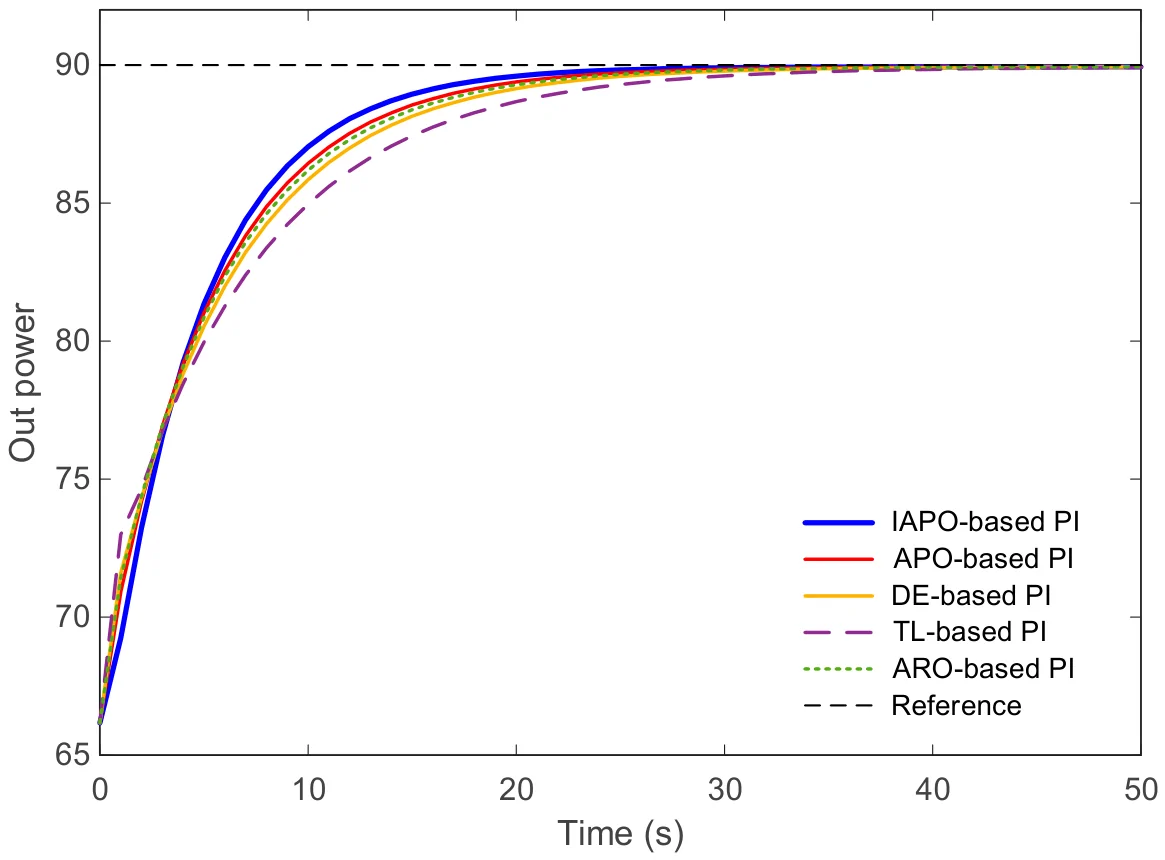
Closed-loop released-thermal-power responses obtained using different PI-tuning methods.

**Figure 8:**
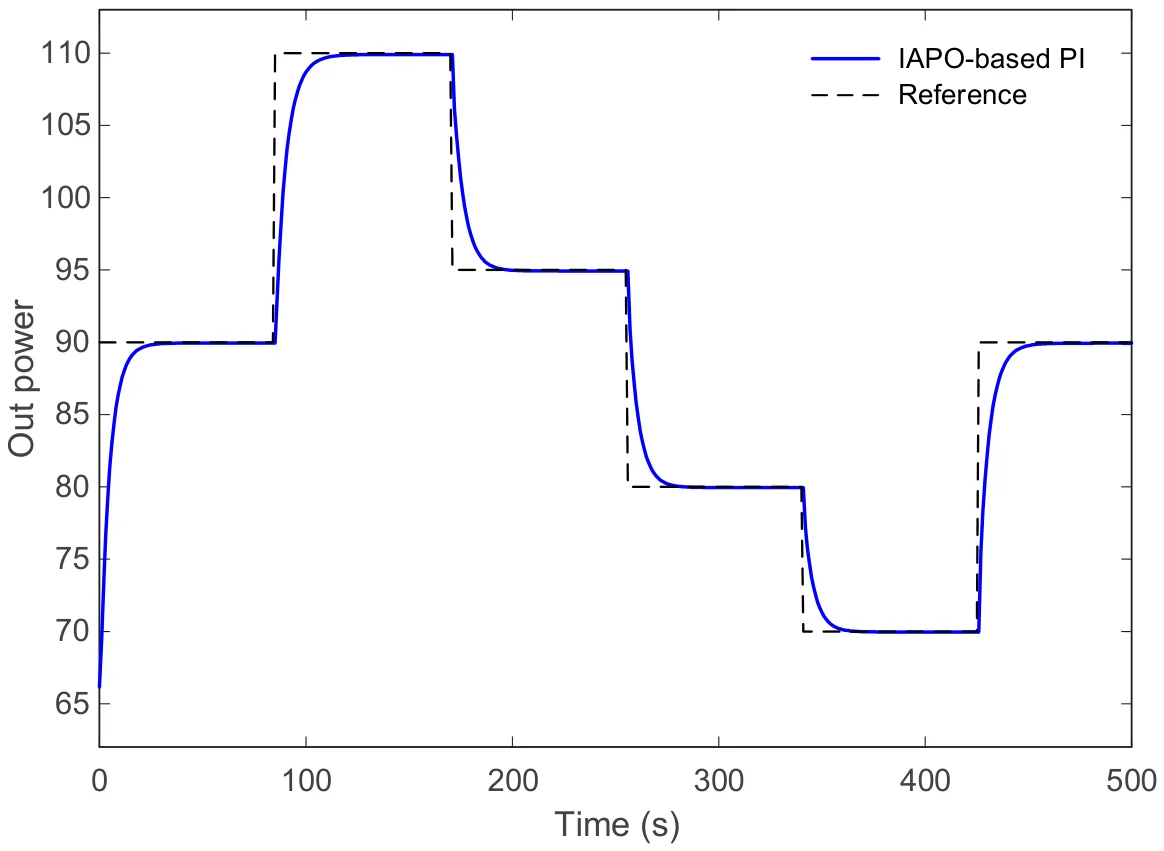
Released-thermal-power tracking of the IAPO-based PI controller under varying reference values.

**Figure 9:**
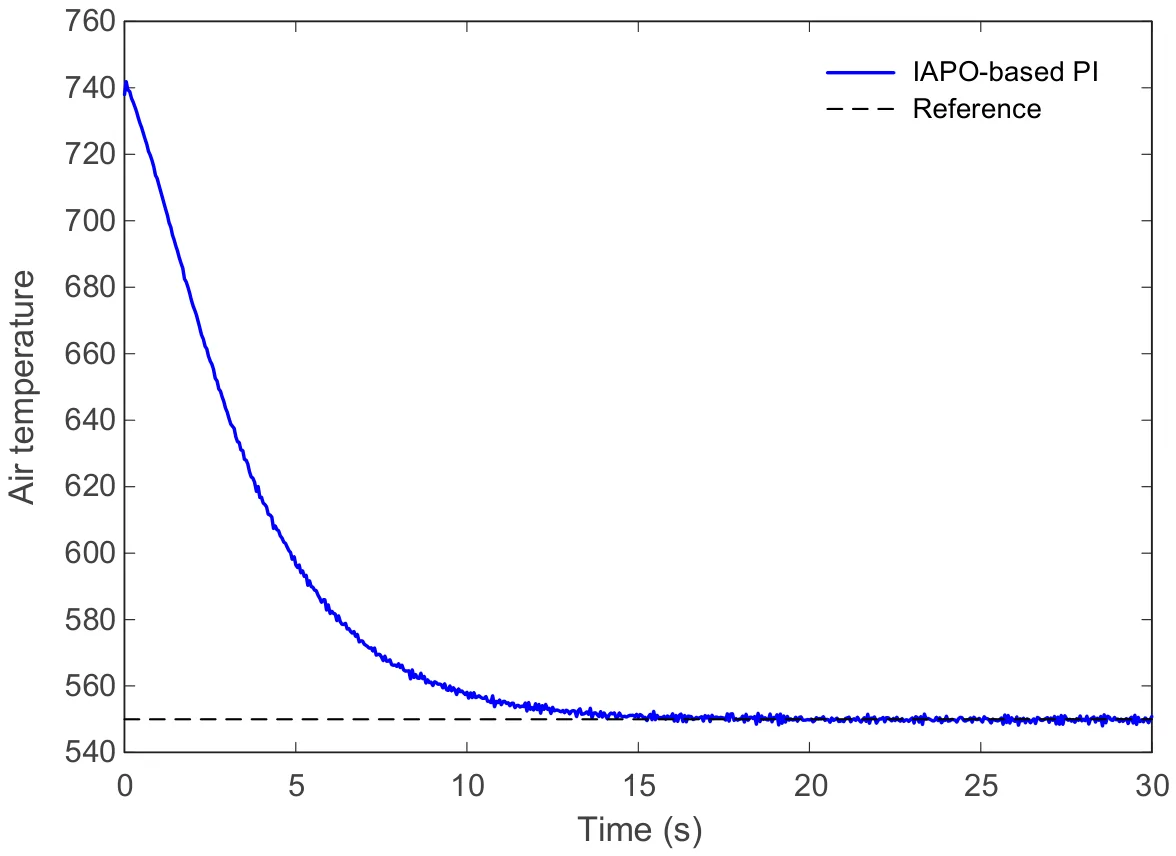
Closed-loop outlet-temperature response of the IAPO-based PI controller in the presence of band-limited measurement noise.

**Table 1:** Main physical and thermophysical parameters of the PCM storage module adopted from [5,33].

Parameter	Symbol	Value
Number of tubes	$N_{tube}$	124
Internal air-channel diameter	$d_{i}$	0.024 m
Tube length	$L_{tube}$	1.2 m
Effective heat-transfer area	A	0.09 m^2^
PCM mass per tube	$m_{PCM}$	7.46 kg
Steel mass per tube	$m_{tube}$	12.34 kg
PCM specific heat capacity in the latent-regime model	$c_{PCM,l}$	1.2 kJ kg^−1^K^−1^
PCM specific heat capacity in the sensible regime	$c_{PCM,s}$	1.1 kJ kg^−1^K^−1^
Steel specific heat capacity	$c_{tube}$	0.5 kJ kg^−1^K^−1^
PCM phase-change enthalpy	$H_{PCM}$	190 kJ kg^−1^
Mean module temperature at the start of liquid–solid phase change	$T_{sp}$	680 K
Mean module temperature at the end of liquid–solid phase change	$T_{ep}$	650 K
Switching temperature between the latent and sensible model states	$T_{cut}$	650 K
Air density	$\rho_{air}$	1.29 kg m^−3^
Reference air specific heat used in the outlet-temperature relation	$c_{p,0}$	1.004 kJ kg^−1^K^−1^
Outer PCM-tube diameter and wall thickness	$D_{tube}$	88 mm × 4 mm
Transverse tube spacing	$S_{T}$	140 mm
Longitudinal tube spacing	$S_{L}$	70 mm
Overall module dimensions	L×W×H	1400 × 980 × 1200 mm

**Table 2:** Identified coefficients of the nonlinear discharging model.

Parameter	Latent Regime	Sensible Regime
${\left(SF\right)}_{j}$	10.409 × 10^−3^	6.6538 × 10^−3^
$r_{1,j}$	−61.9661	−56.4071
$r_{2,j}$	0.644398	Determined from the transition balance at 650 K

**Table 3:** Statistical comparison of ITAE values for outlet-temperature controller tuning.

Measure	IAPO	APO	DE	ARO
Best	1.7743 × 10^3^	1.8793 × 10^3^	2.1711 × 10^3^	1.8929 × 10^3^
Worst	1.8857 × 10^3^	2.0144 × 10^3^	2.3218 × 10^3^	1.9692× 10^3^
Mean	1.8165 × 10^3^	1.9348 × 10^3^	2.2432 × 10^3^	1.9253 × 10^3^
Standard deviation	31.2287	42.1903	38.7968	19.2418
*p*-value vs. IAPO	—	1.2290 × 10^−5^	1.2290 × 10^−5^	1.2290 × 10^−5^
$\mathrm{Cohen}’s\;\left|d\right|$ vs. IAPO	—	3.19	12.12	4.19
Computation time (s)	190.45	187.93	217.41	192.08

**Table 4:** PI controller gains obtained for outlet-temperature regulation.

Parameter	IAPO	APO	DE	TL	ARO
$K_{P}$	−0.8149	−0.7561	−0.8067	−0.9497	−0.9631
$K_{I}$	−0.7974	−0.7683	−0.7226	−0.7130	−0.7923

**Table 5:** Time-domain performance indices for outlet-temperature regulation.

Stability Metric	IAPO	APO	DE	TL	ARO
$t_{r}$(s)	3.6647	3.7205	4.4241	5.0117	4.3110
$t_{s}$(s)	7.1417	7.2537	8.4513	9.5269	8.1608
OS (%)	0.0899	0.0947	0.1031	0.1050	0.0907

**Table 6:** PI controller gains obtained for released-thermal-power regulation.

Parameter	IAPO	APO	DE	TL	ARO
$K_{P}$	0.5432	0.8375	0.9589	1.2042	0.9251
$K_{I}$	0.7791	0.7479	0.7023	0.6384	0.7310

**Table 7:** Time-domain performance indices for released-thermal-power regulation.

Stability Metric	IAPO	APO	DE	TL	ARO
$t_{r}$(s)	10.2504	11.7172	13.0022	15.2080	12.2847
$t_{s}$(s)	19.0326	21.5541	23.9464	28.2615	22.6148
OS (%)	0	0	0	0	0

## Data Availability

The data presented in this study are available on request from the corresponding author.

## Abbreviations

The following abbreviations are used in this manuscript:

AI	Artificial Intelligence
APO	Artificial Protozoa Optimizer
ARO	Artificial Rabbits Optimization
DE	Differential Evolution
IAPO	Improved Artificial Protozoa Optimizer
ITAE	Integral of Time-Weighted Absolute Error
MPC	Model Predictive Control
PCM	Phase Change Material
PI	Proportional–Integral
PID	Proportional–Integral–Derivative
TES	Thermal Energy Storage
TL	Tyreus–Luyben
